# Delayed Diagnosis of Gastric Outlet Obstruction from Bouveret Syndrome in a Young Woman

**DOI:** 10.5811/westjem.2014.10.23049

**Published:** 2014-11-10

**Authors:** Zachary Smith, Jodie Totten, Adrienne Hughes, Jared Strote

**Affiliations:** * Madigan Army Medical Center, Department of Emergency Medicine, Tacoma, Washington; † University of Washington-Seattle Campus, Division of Emergency Medicine, Seattle, Washington

## Abstract

Bouveret syndrome is a rare presentation of gastric outlet obstruction caused by a gallstone in the proximal duodenum via a bilioenteric fistula. This is an infrequent although clinically significant cause of abdominal pain, almost exclusively in the elderly. The clinical presentation is similar to that of a small bowel obstruction with abdominal pain, nausea and vomiting. Surgery or endoscopy is often required for definitive diagnosis and therapy. We describe the case of a young woman with this condition who had a delayed diagnosis in part because of her age and the rarity of the condition.

## INTRODUCTION

Bouveret syndrome is a rare presentation of gastric outlet obstruction caused by a gallstone in the proximal duodenum via a bilioenteric fistula. Gallstone ileus is uncommon itself, accounting for only 1–4% of intestinal obstructions and Bouveret syndrome is seen in only 1–3% of gallstone ileus cases. Presentations of Bouveret syndrome are at risk of being missed, as symptoms are frequently non-specific and presentations are often benign. The disease is almost exclusively one of the elderly, increasing the likelihood of missed diagnosis when it occurs in younger patients.^1^ We describe the case of a young woman with this condition who had a delayed diagnosis in part because of her age and rarity of the condition.

## CASE REPORT

A 26-year-old female presented to our emergency department (ED) complaining of abdominal pain. The pain was located in the right upper quadrant; it was described as severe, achy, constant, non-radiating, and associated with nausea and non-bilious, non-bloody vomiting. She had experienced intermittent pain and vomiting for several months but had not sought medical attention until the day of presentation. She denied any fever, chills, hematemesis, diarrhea, melena, pruritis, abnormal vaginal bleeding or discharge. She had a chronic hepatitis C virus infection and a history of prior intravenous heroin use but was currently on methadone maintenance therapy. She smoked approximately 5–10 cigarettes per day and denied any active drug or alcohol use. She did not take any medications and had no allergies.

On examination, her vital signs were all within normal limits. The patient had moderate abdominal tenderness to palpation in the right upper quadrant and mid-epigastrium and an equivocal Murphy’s sign. The remainder of her examination was notable for a lack of abdominal distension, rebound, guarding or any palpable masses. Laboratory studies demonstrated a slight transaminitis (aspartate aminotransferase [AST] 82 [normal range 15–40] units/L, alanine aminotransferase [ALT] 84 [normal range 6–40] units/L, and alkaline phosphatase 135 [normal range 25–100] units/L). Blood glucose, lipase, bilirubin, hematocrit, white blood count, electrolytes, renal function and coagulation function tests were all normal, and urine pregnancy test was negative. An abdominal plain film showed no free air and normal bowel gas pattern. An abdominal ultrasound revealed no biliary ductal dilation, although the gallbladder was unable to be visualized. Repeat evaluations after treatment with aluminum and magnesium hydroxide demonstrated a benign abdomen with improved symptoms. Omeprazole and odansetron were prescribed and the patient was discharged home.

Three days later, the patient returned to the ED. She reported worsening abdominal pain and increased nausea despite taking the prescribed medications. On exam, her vitals were still normal. She again demonstrated moderate right upper quadrant and mid-epigastric tenderness without distention, masses, Murphy’s sign, guarding, or rebound. The remainder of the examination was unchanged and normal. Laboratory studies revealed an AST of 252 units/L, ALT of 272 units/L, and alkaline phosphatase of 133 units/L. The patient’s urine pregnancy test, lipase, bilirubin, hematocrit, white blood count, electrolytes, renal function and coagulation function tests remained within normal limits. A computed tomography (CT) of the abdomen/pelvis was obtained and demonstrated mild thickening of the gallbladder wall, circumferential wall thickening and mucosal enhancement of the first part of the duodenum, with surrounding omental stranding and an associated visualized fistulous tract to the gallbladder. There were no gallstones identified in the bowel lumen and no clear evidence of gastric obstruction (Figure).

Despite the lack of radiologic evidence of obstruction, the patient was admitted to the general surgery service with the clinical diagnosis of presumed gastric outlet obstruction likely secondary to proximal gallstone ileus. A nasogastric tube was placed to decompress the stomach. Initial treatment included administration of intravenous fluids, anti-emetics, and pain medications.

During her admission, the patient underwent fluoroscopic examination of the duodenum with gastrograffin which demonstrated complete obstruction between the duodenum and gastric antrum. Subsequent esophagogastroduodenoscopy (EGD) demonstrated a large gallstone occluding the lumen of the pylorus and the patient was subsequently scheduled for endoscopic removal of gallstone impaction the following day under general anesthesia. Repeat EGD demonstrated severe inflammation at the level of the pylorus and a fistulous tract between the gallbladder and duodenum consistent with cholecystoduodenal fistula. The previously noted gallstone was no longer present. Serial balloon dilation was performed with partial but incomplete resolution of the duodenal bulb gastric outlet obstruction. A nasojejunal feeding tube was placed, and the patient was started on enteral nutrition. Her diet was slowly advanced and no further interventions were performed.

## DISCUSSION

Bouveret syndrome is named after Leon Bouveret who published two case studies of this condition in 1896.^1^ It is defined as gastric outlet obstruction by a gallstone in the duodenum, which occurs via gallstone erosion through the intestinal wall. This creates a bilioenteric fistula, most commonly cholecystoduodenal.^2^ The proposed mechanism involves chronic inflammation leading to intra-abdominal adhesions as well as acute cholodocolithiasis. This, in turn, creates increased pressure and ultimately gallbladder ischemia, necrosis, and subsequent bilioenteric fistula.^2^ Duodenal diverticula may also place patients at higher risk.^3^

Bouveret syndrome occurs more frequently in females than males, which is consistent with the higher frequency of gallstones in women.^3^ It is overwhelmingly a disease of the elderly; a recent review, which examined all published reports of Bouveret syndrome from 1974 (128 patients), described a mean age of 71 years with a standard deviation of 11.

Patients with Bouveret syndrome present with symptoms similar to small bowel obstruction: nausea, vomiting and epigastric abdominal pain, often waxing and waning with resolution of symptoms intermittently, making diagnosis particularly challenging. Common exam findings include abdominal tenderness, dehydration, and abdominal distention.^1^ Hematemesis may be present due to erosion of the cystic artery and fever is not uncommon.^1^,^2^

Abdominal plain radiographs are often the first imaging study obtained and may illustrate Rigler’s triad of gallstone ileus of ectopic gallstones, pneumobilia, and small bowel obstruction.^4^ Plain films, however, are diagnostic of Bouveret’s syndrome in only approximately 21% of patients, and further imaging usually occurs.^3^ Ultrasound can be helpful to show biliary pathology, but ultimately CT is needed to confirm the diagnosis of obstruction and stone, even though a fistula is rarely visualized on CT.^1^,^3^,^5^ Endoscopy is an appealing combined diagnostic and therapeutic option as these patients frequently have multiple co-morbidities and the obstructing stone is seen in 69% of endoscopies; however, removal is only successful in 10%.^1^,^3^ Various types of lithotripsy procedures have also been used. Up to 91% of patients may ultimately require surgical repair;^3^ enterolithotomy or gastrotomy are the most common options, frequently including a cholelcystectomy.^1^ The mortality rates are now estimated at 12%, in great part due to the advanced age and frequent co-morbidities of the patients who develop this condition.^3^

Although rare, and generally thought of as a disease of the elderly, we suggest that Bouveret syndrome be considered in younger patients presenting with signs and symptoms suggestive of small bowel obstruction but without a clear cause.

## Figures and Tables

**Figure:**
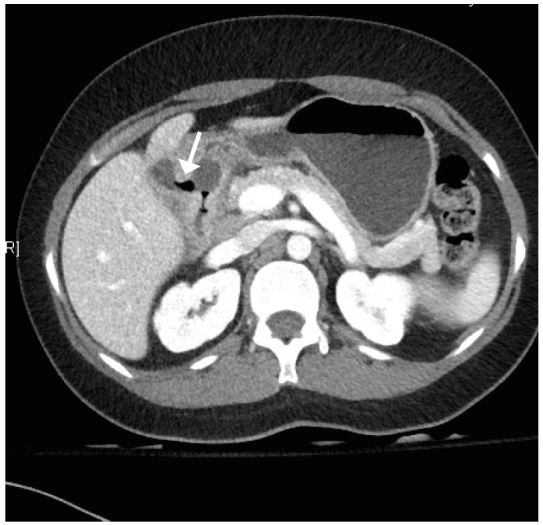
Transverse computed tomography of air passing from duodenum through a fistula into the gallbladder.
